# Developmental mechanisms underlying differential claw expression in the autopodia of geckos

**DOI:** 10.1186/s13227-015-0003-9

**Published:** 2015-04-10

**Authors:** Eraqi R Khannoon, Anthony P Russell, Abigail S Tucker

**Affiliations:** Zoology Department, Faculty of Science, Fayoum University, Fayoum, 63514 Egypt; Department of Biological Sciences, University of Calgary, 2500 University Drive NW, Calgary, Alberta T2N 1N4 Canada; King’s College London, Floor 27 Guy’s Tower, Guy’s Hospital, Great Maze Pond, London Bridge, London, SE1 9RT UK

**Keywords:** *Tarentola*, Apoptosis, Regression, Proliferation

## Abstract

**Background:**

The limb and autopodium are frequently employed to study pattern formation during embryonic development, providing insights into how cells give rise to complex anatomical structures. With regard to the differentiation of structures at the distal tips of digits, geckos constitute an attractive clade, because within their ranks they exhibit multiple independent occurrences of claw loss and reduction, these being linked to the development of adhesive pads. The developmental patterns that lead to claw loss, however, remain undescribed. Among geckos, *Tarentola* is a genus characterized by large claws on digits III and IV of the manus and pes, with digits I, II, and V bearing only vestigial claws, or lacking them entirely. The variable expression of claws on different digits provides the opportunity to investigate the processes leading to claw reduction and loss within a single species.

**Results:**

Here, we document the embryonic developmental dynamics that lead to this intraspecifically variable pattern, focusing on the cellular processes of proliferation and cell death. We find that claws initially develop on all digits of all autopodia, but, later in development, those of digits I, II, and V regress, leading to the adult condition in which robust claws are evident only on digits III and IV. Early apoptotic activity at the digit tips, followed by apoptosis of the claw primordium, premature ossification of the terminal phalanges, and later differential proliferative activity are collectively responsible for claw regression in particular digits.

**Conclusions:**

Claw reduction and loss in *Tarentola* result from differential intensities of apoptosis and cellular proliferation in different digits, and these processes have already had some effect before visible signs of claw development are evident. The differential processes persist through later developmental stages. Variable expression of iteratively homologous structures between digits within autopodia makes claw reduction and loss in *Tarentola* an excellent vehicle for exploring the developmental mechanisms that lead to evolutionary reduction and loss of structures.

**Electronic supplementary material:**

The online version of this article (doi:10.1186/s13227-015-0003-9) contains supplementary material, which is available to authorized users.

## Background

The tips of the digits of amniote vertebrates are governed by a molecular developmental program distinct from that which controls their more proximal portions [1,2]. Digit tip formation takes place when FGF (fibroblast growth factor) signaling ceases in the apical ectodermal ridge (possibly in response to initiation of Wnt signaling), and a secondary signal takes over [1]. Candidate genes involved in the digit tip program have been identified (see Casanova *et al*. [2] for a review), including *bone morphogenetic proteins* (*BMPs*), *BAMBI, noggin*, and the *Wnt* pathway [3-6]. As end organs, amniote digit tips are primitively endowed with a keratinous claw, although modifications of this are also expressed as nails and hooves in some mammals.

The claw, in both reptiles and mammals, begins as a thickening of the overlying epithelium, with a fold developing at its proximal edge [7,8]. In lizards, the keratinous sheath of the claw has been described as being a modified terminal scale [9] that expresses a greater intensity and earlier onset of keratinization than do other scales of the digit. As claw development proceeds, the distalmost non-ungual dorsal scale grows over the fold, forming a hinge region, thereby internalizing the claw primordium [8]. This step is followed by the production of keratins from stratified keratinocytes [8]. Thymidine labeling indicates that proliferation occurs along the whole length of the claw epidermis [8]. Greater proliferation of the unguis, comprised of tough β-keratins [9], relative to the sub-unguis, composed of more pliable α-keratins, has been suggested to generate the curvature of the claw [10].

The absence of a keratinized integumentary capping at the distal end of the digits is of sporadic occurrence throughout amniotes and may be associated with modified functional roles of the digit tips. Such absence may apply to all digits, or may be regionally evident, with some, but not all, digits of an autopodium, exhibiting this state. There may even be differences between positionally equivalent digits of the manus and pes.

Within the Gekkota, multiple occurrences of claw reduction and loss are evident, its prevalence being much higher than in any other lineage of normal-limbed lizards (those not exhibiting patterns of limb reduction) [11]. All of the instances within the Gekkota occur in taxa that possess a subdigital adhesive system. The finer details of how claws are used during locomotion are yet to be elucidated [12], but they are employed, in some circumstances, to interact with asperities in rough surfaces to promote grip [12,13]. They are also used in digging, grasping of conspecifics (as in mating), and may even be involved in the manipulation of eggs at the time of oviposition, when the hind feet are used to roll the eggs on the ground to coat them with particles [14] or to place them into contact with surfaces to which they stick [15].

Mapping the instances of claw reduction and loss (Russell AP: The foot of gekkonid lizards: a study in comparative and functional anatomy. Unpublished PhD. Thesis, 1972) within the Gekkota onto the phylogeny published by Gamble *et al*. [16] reveals ten separate occurrences. Seven of these occur within the Gekkonidae: (1) In the *Pachydactylus* radiation (*Pachydactylus*, *Elasmodactylus*, *Chondrodactylus*, C*olopus*, *Rhoptropus*) all taxa exhibit either complete absence of claws on all digits or their reduction to vestigial, needle-like structures. The manus is generally entirely clawless (except in some digging species of *Pachydactylus*, in which minute claws are present), and the pes generally carries vestigial, needle-like claws (the presence of which is in some cases restricted to digits III and IV), which may be sexually dimorphic (being larger and more robust in females). (2) In the clade containing *Homopholis*, *Blaesodactylus*, and *Geckolepis*, these three genera bear robust claws on digits II to V of the manus and pes, whereas digit I carries a minute claw or is clawless. Within a species, manual digit I may lack a claw while pedal digit I may display a minute one. (3) In *Phelsuma* (including *Rhoptropella*), all digits are clawless or carry vestigial, needle-like claws. Digit I, manus and pes, is itself vestigial in this taxon. (4) In *Ebenavia*, all digits are clawless. (5) In the *Gekko* clade (*Gekko*, *Pseudogekko*, *Luperosaurus*, *Ptychozoon*, *Lepidodactylus*, *Hemiphyllodactylus*, *Gehyra*, *Perochirus*), digits II to V of the manus and pes carry robust claws. Digit I of the manus and pes may bear a minute claw or exhibit no presence of this. In *Perochirus*, diminution of the claw on manual and pedal digit I is accompanied by vestigialization of the digit itself. (6/7) Although the genus *Hemidactylus* generally displays robust claws on all digits, two species, *H. greefii* and *H. brasiliana*, independently show reduction of the claw to a small structure in manual digit I, and pedal digit I is also clawless in the latter.

Within the Phyllodactylidae, (8) *Tarentola* is characterized by having digits III and IV of the manus and pes strongly clawed, whereas digits I, II, and V of the autopodia carry either minute, needle-like claws (pes) or lack them entirely (manus). In some species, pedal digit I is also entirely without a claw.

Within the Diplodactylidae, there are two independent instances of claw reduction. (9) In *Crenadactylus*, all digits are clawless. (10) In *Pseudothecadactylus*, digit I of the manus and pes lacks a claw while all other digits are robustly clawed.

These multiple instances of claw reduction and loss are suggestive of functional differences between digits [17] and furnish the opportunity for investigating developmental mechanisms by which claw reduction and loss come about. The phyllodacylid genus *Tarentola* presents a particularly attractive vehicle for this because it exhibits the retention of fully developed claws on digits III and IV of the manus and pes alongside vestigialization or complete absence of claws on adjacent digits. This taxon thus permits the investigation of the development of full claw expression, vestigialization, and complete absence within and between autopodia of a single species. Despite our knowledge of the incidence of claw loss in geckos, an understanding of the developmental patterns that lead to claw loss have not been described.

We herein explore the timing and interaction of developmental processes bringing about full expression, reduction, or complete absence of the keratinized claw sheath in *Tarentola annularis.* In doing so, we also explore developmental processes in the underlying ungual phalanx. We predict that all digits will exhibit identical initial stages of claw formation that commence at the same time on all digits, and that regression of claw sheath expression and regression of the underlying ungual phalanx will subsequently occur in those digits that ultimately exhibit only minute claws or are completely devoid of them. In relation to this, we hypothesize that apoptosis (programmed cell death) plays an important role in the reduction and elimination of the claw sheath rudiments and portions of the underlying phalanx, as it does in the removal of additional tooth placodes in the mouse [18], the removal of non-functional tooth germs in the gecko *Paroedura* [19], and in the reduction of whole digits in some mammals [20]. Furthermore, we hypothesize that apoptosis will be accompanied by a reduction in proliferation of generative cells in the claw sheath and ungual phalanx, as has been noted in the vestigialization of murine tooth germs and in loss of digits in skinks [21-23]. The alternative to the processes outlined above is that those digits displaying claw reduction or loss do so by initiating the developmental processes leading to claw sheath formation late or not at all. By understanding the mechanisms by which complex structures are lost, we aim to provide an insight into how variation can be generated during evolution, allowing for adaptation to specialized functions.

## Methods

### Specimens

For gross morphological, histological, and immunohistochemical observations, adult, juvenile, and embryonic specimens of *Tarentola annularis* were assembled. For gross morphological observations, a series of juvenile (*N* = 9) and adult (*N* = 12) individuals were obtained from breeders in Egypt, sacrificed, fixed in neutral buffered formalin, and stored in 70% ethanol. To obtain embryos, gravid female *T. annularis* (*N* = 37) were maintained in laboratory cages and eggs, when laid, were carefully collected and transferred to plastic boxes containing perlite and incubated at a constant temperature of 30°C and a humidity of 85% to 90%. All animals were treated according to the refinement principles of respect, care, and minimization of suffering, and all work was conducted after being approved by the Committee on the Ethics of Animal Experiments, Zoology Department, Faculty of Science, Fayoum University. Euthanasia protocols used were conducted according to ethical concepts of diminishing animal pain, as laid out by the UK Home Office.

Embryos at different developmental stages were removed from their eggs and sacrificed by decapitation after cooling on ice. Digits from selected developmental stages (Additional file 1: Table S1) were chosen for examination: for ease of comparison, the Hamburger and Hamilton [24] embryonic stage equivalent for squamates [25] is provided. Digits that, in the adult state, bear fully developed, reduced or no claw sheaths were compared employing the techniques outlined below.

### Morphology

Manūs (*N* = 9) and pedes (*N* = 9) of ethanol-preserved juveniles and adults of *T. annularis* and of various embryonic stages (*N* = 3 to 5 for each stage) were examined using a Nikon 50i microscope and Nikon DS-5 M camera head and camera control unit (Nikon, Tokyo, Japan) and a Leica stereo microscope (MZFiii) and Leica DFC300 camera (Leica, Solms, Germany).

### Scanning electron microscopy

Samples of manūs and pedes of ethanol-preserved selected embryonic stages (Additional file 1: Table S1) of *T. annularis* (*N* = 2 for each of stages 35, 36, 37, and 38) were dehydrated using graded series of ethanols, culminating with immersion in 100% ethanol (2× 30 min); placed in specimen capsules containing 100% ethanol and critical-point dried; mounted on aluminum stubs using copper double-sided tape and silver conductive paint, and coated with gold/palladium (20-nm thick film) in a Polaron SC515 SEM coating system. Specimens were examined in a JEOL6400 SEM (JEOL Ltd., Akishima-shi, Japan).

### Histology

Samples of clawed, reduced-clawed, and unclawed digits were processed for histology (stages 35, *N* = 7; stage 36, *N* = 7; stage 37, *N* = 5). Following fixation in 10% neutral buffered formalin, samples were washed in distilled water and then in phosphate-buffered saline (PBS) and subsequently transferred to 70% ethanol. They were then dehydrated by taking them through a graded series of ethanols, culminating in three changes of 100% ethanol (Sigma-Aldrich, St. Louis, MO, USA), cleared in three changes of Histoclear (National Diagnostics, Atlanta, GA, USA), and infiltrated with three to four changes of molten paraffin wax (Thermo Scientific Raymond Lamb, Thermo Fisher Scientific, Waltham, MA, USA) (57°C) prior to embedding. Frontal and sagittal sections of 8 μm were cut on a Leica microtome (RM2245). Sections were subjected to trichrome staining (sirus red, alcian blue, Ehrlich’s hematoxylin) [26], and the stained sections were mounted with DPX (BDH). Sections were viewed on a Nikon Eclipse 80i microscope and photographed using a Nikon Digital Sight Camera.

### PCNA proliferation

In order to view proliferating cells, immunohistochemistry for PCNA (proliferating cell nuclear antigen) was performed on paraffin wax sections (stage 36, *N* = 7; stage 38, *N* = 2) that had been dewaxed in Histoclear and dehydrated through an ethanol series to PBS. PCNA detects cells in G1, S, G2, and M phase. Slides were immersed in proteinase K 20 μg/ml in PBS for 15 min and subsequently treated in 3% H_2_O_2_ for 10 min. An antigen retrieval step was performed using pH6 0.01 M citric acid at 95°C for 5 to 10 min. Samples were left to cool, washed in PBS, and then permeabilized in 0.05% trypsin in PBS. PCNA staining was then carried out using a PCNA labeling kit (Life Technologies 93–1143, Life Technologies, Carlsbad, CA, USA) following the manufacturer’s instructions. The color reaction was performed using a DAB kit (Vector Labs, Burlingame, CA, USA) following the manufacturer’s instructions. Samples were washed in PBS before being counterstained with 1% alcoholic eosin (Sigma) for 30 s, washed in 90%, 95%, and 100% ethanol, and mounted in DPX (BDH). Sections were viewed on a Nikon Eclipse 80i microscope and photographed using a Nikon Digital Sight Camera.

### TUNEL

In order to identify cells undergoing programmed cell death, TUNEL (terminal deoxynucleotidyl transferase dUTP nick end labeling) staining was employed. Paraffin wax sections (stage 36, *N* = 7) were dewaxed in Histoclear and rehydrated in decreasing ethanol concentrations, then washed with PBS. They were then immersed in freshly diluted protein digestion enzyme (Proteinase K; 65 μl/5 cm^2^) for 15 min. Following this, they were washed in dH_2_O, then quenched in 3% H_2_O_2_ in PBS for 5 min at RT, then washed in two or three changes of PBS. A TUNEL apoptosis detection kit (Millipore S7100, Millipore, Billerica, MA, USA) was used to label cells following the manufacturer’s instructions. A 75 μl/5 cm^2^ of equilibrium buffer was then applied for at least 10 s at RT. A 55 μl/5 cm^2^ of working strength TDT enzyme was then pipetted onto the sections, and they were incubated in a humidified chamber for 1 h at 37°C. Following this, a stop/wash buffer was applied for 10 min at RT. The sections were then washed in three changes of PBS, and subsequently 65 μl/5 cm^2^ of anti-digoxignenin conjugate was applied for 30 min in a humidified chamber at RT. The color reaction, counterstaining, mounting, and photographing were as for PCNA (above).

### Caspase-3 immunofluorescence

To identify cells early on in the apoptotic pathway, localization of activated caspase 3 was investigated. Paraffin wax sections (stage 35, *N* = 6) were dewaxed in Histoclear, rehydrated in decreasing ethanol concentrations (as above), and then washed in PBS. They were then subjected to an antigen retrieval wash for 30 min in Tris-EDTA buffer (pH 9.0) at 95°C. Subsequently, the samples were left for 90 min to cool to RT and were then subjected to a 10-min wash in PBS, followed by 20 min in PBS with 0.5% Triton® X-100 (Sigma-Aldrich, T9284). Slides were then transferred to a covered moist humidified chamber, and 150 μl of blocking solution (10% goat serum and 1% BSA in PBS with 0.1% Triton® X-100) was added to each slide and left for 1 h at RT. Sections were then incubated overnight at 4°C with activated rabbit caspase-3 primary antibody (1 in 200) in blocking solution in the humidified chamber. Three 1-h washes in PBS were then undertaken, followed by incubation for 1 h with Alexa-fluor 568 goat anti-rabbit (1 in 500) in blocking solution (as above) at RT in the humidified chamber. Sections were then subjected to six 5-min washes in PBS in a Coplin jar wrapped in aluminum foil to omit light. Sections were mounted with Fluoroshield™ with DAPI (Sigma-Aldrich) mounting medium, cover slipped, and imaged on a Zeiss photomicroscope (Axioskop 2) (Zeiss, Jena, Germany).

### DAPI

Nuclear staining using DAPI was employed to visualize the nuclei in the developing claw. Parraffin wax sections (stage 35, *N* = 5; stage 36, *N* = 4) were dewaxed, rehydrated, and mounted with Fluoroshield™ with DAPI (Sigma-Aldrich) mounting medium and cover slipped. Slides were placed in a lightproof, dry chamber and stored at 4°C prior to photographing with a Zeiss fluorescence microscope (Axioskop 2).

## Results

### Claw distribution in *Tarentola annularis*

**Figure 1:**
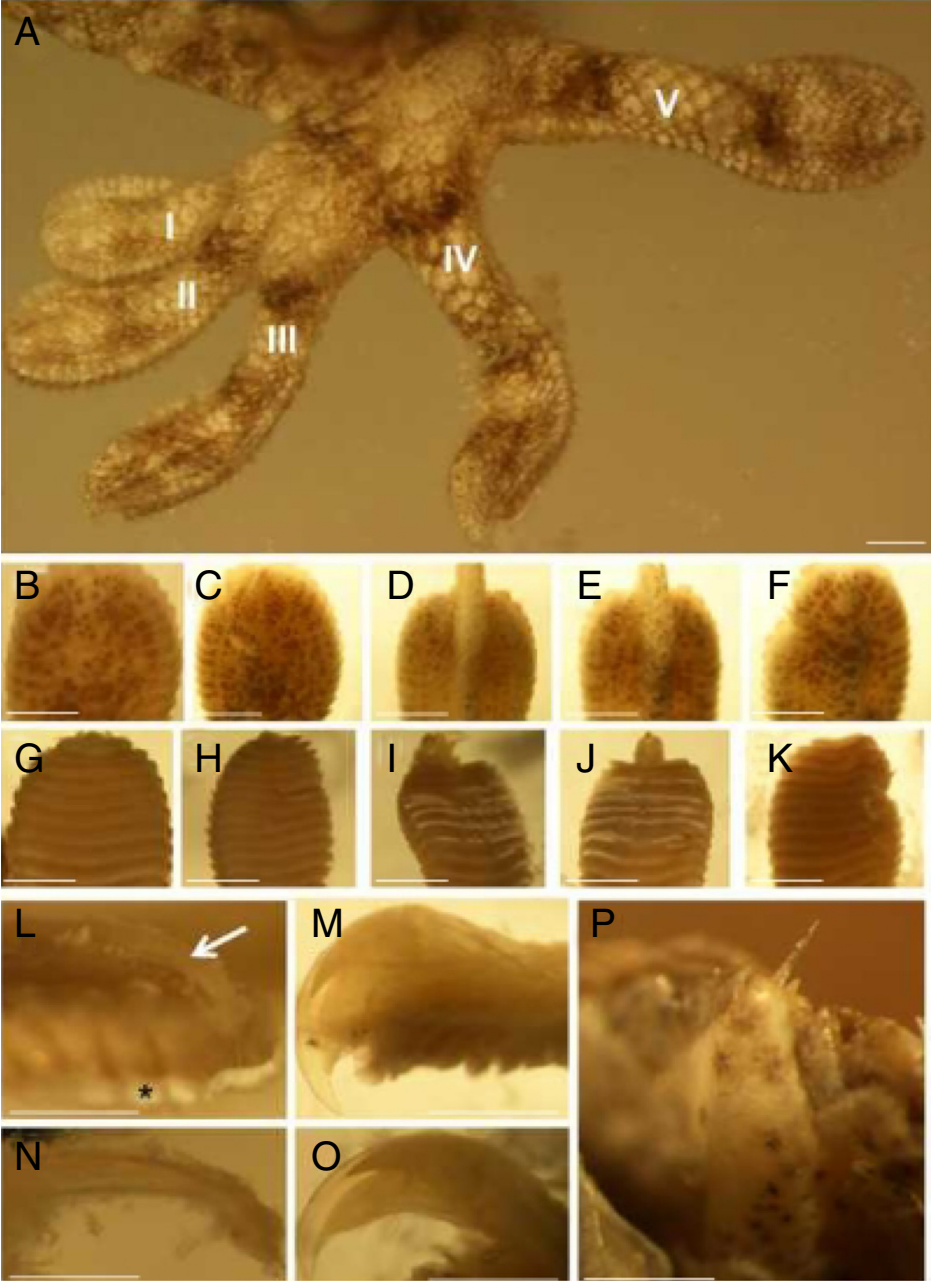
**Digit form in adult** ***Tarentola annularis*** **. (A)** Dorsal view of the left pes. Roman numerals I to V indicate the first to fifth digits, respectively. Note the raised penultimate phalanx that forms a longitudinal ridge on the distal ends of digits III and IV. This carries a large recurved claw that tips the ungual phalanx of each of these digits. Digits I, II, and V lack this ridge, and no claw is evident on these digits. **(B-F)** Dorsal aspect of, from left to right, pedal digits I to V. Note the raised ridge of the penultimate phalanx on digits III and IV that carries the large ungual phalanx and claw beyond the distal extremity of the subdigital adhesive pad. Digits I, II, and V (b, c, and f, respectively) lack this ridge. **(G-K)** Ventral aspect of, from left to right, pedal digits I to V, showing the large claw that extends beyond the distal extremity of the adhesive pad on digits III and IV, and the absence of a visible claw on digits I, II, and V. **(L)** Lateral view of the distal extremity of pedal digit II with the skin removed, revealing the elongate, gently curved ungual phalanx (arrow) supporting the distal end of the adhesive pad (setal fields denoted by an asterisk). **(M)** Lateral view of the distal extremity of pedal digit III with the skin removed, displaying large, strongly-curved claw that extends distally beyond the adhesive pad. **(N)** Lateral view of ungual phalanx of pedal digit II with the surrounding soft tissue removed. **(O)** Lateral view of the distal ungual phalanx and claw sheath of pedal digit III with the surrounding soft tissue removed. **(P)** Needle-like claw present on digit II of the pes. Scale bars, 1 mm.

Examination of the digits of *Tarentola annularis* confirmed that in adults, fully developed claws are present only on digits III and IV of the manus and pes (Figure 1A,D,E,I,J). No claws were evident on digits I, II, and V of the manus, but vestigial, needle-like claws were present on these digits of the pes (Figure 1P). The terminal phalanges of the clawless and reduced-clawed digits were elongated and slender (Figure 1L,N) rather than being robust, tall, and markedly curved like the ungual phalanges of the strongly clawed digits (Figure 1M,O).

**Figure 2:**
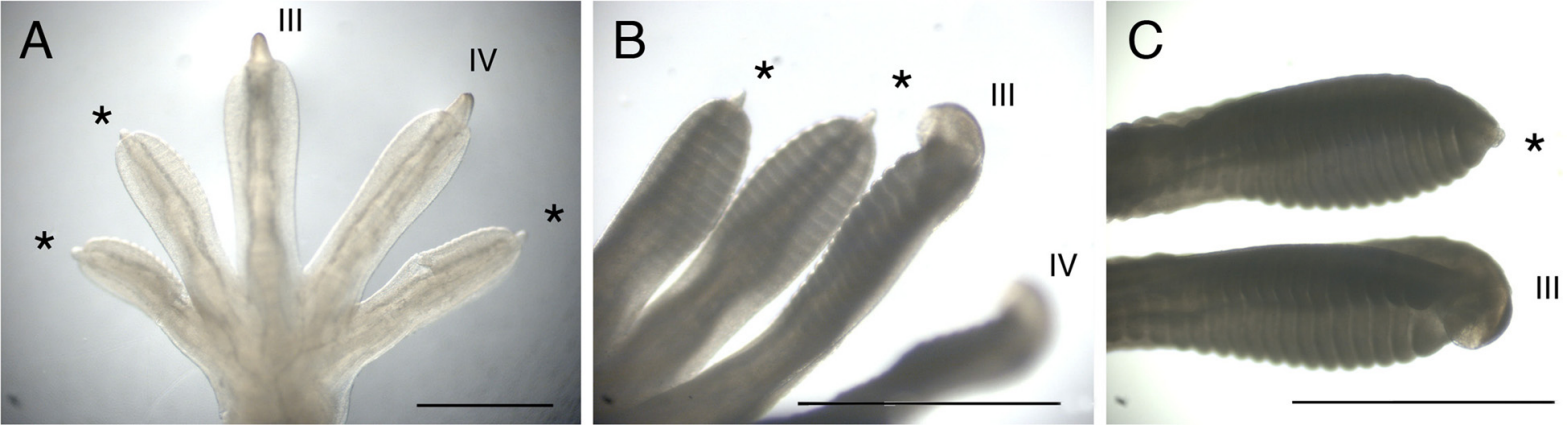
**Appearance of the claws of** ***Tarentola annularis*** **at stages 35 to 37 of development. (A)** The left manus in dorsal view at late stage 35 of development. Claws are evident protruding from the distal tip of all five digits. These are largest in digits III and IV, and smaller in digits I, II, and V (asterisks). **(B)** The left pes in dorsal view at stage 36 of development. The claws on digits I, II (asterisks), and V (not shown) have begun to regress, whereas those on digits III and IV continue to enlarge. **(C)** Dorsal aspect of the right pes at stage 37. By this point only a rudimentary claw is evident on digit II (asterisk), whereas that on digit III is further enlarged. Scale bars, 1 mm.

**Figure 3:**
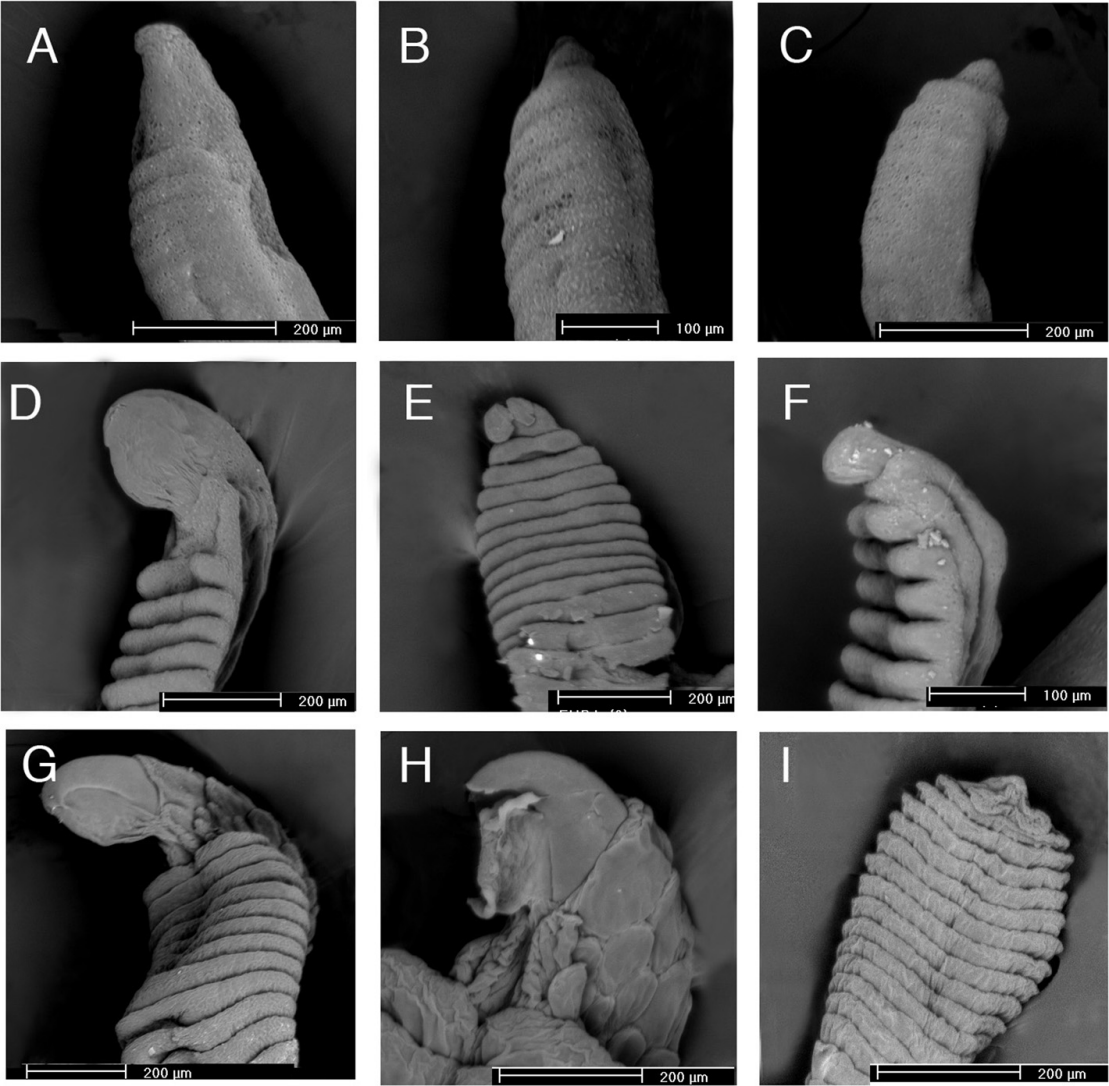
**SEM of claw development in** ***Tarentola annularis*** **. (A-C)** Manual digits at stage 35 of development. A large claw is evident on digit IV of the manus **(A)**, whereas the claw is small on digits V **(B)** and **I** **(C)**. **(D-F)** Pedal digits at stage 36 of development. A large claw is evident on digit III **(D)**, and small claw rudiments are present on digits V **(E)** and **I** **(F)**. **(G)** Manual digit III at stage 37 of development. **(H,I)** Pedal digits at stage 38 of development. The large claw on digit IV **(H)** contrasts with the greatly reduced structure on digit V **(I)**, where only a scale is now evident distally.

During embryogenesis of *T. annularis*, claw sheaths initiated simultaneously on all five digits of both the manus and pes during early development and were associated with a protrusion of the distalmost phalanx that extended beyond the expanding subdigital pad (Figure 2A). However, from the outset, at their earliest appearance (at stage 35), the claw rudiments on the clawless and reduced-clawed digits (I, II, and V) were already noticeably smaller than those on the fullyclawed digits (Figures 2A and 3A,B,C). As the digits continued to develop, the distal extension at the tips of digits I, II, and V became relatively reduced in size whereas those on digits III and IV continued to enlarge (Figures 2B,C and 3D-F). The claws on digits III and IV were pronounced by stage 37 (Figure 3G), and by stage 38 there was no visible evidence of a claw on digits I, II, and V of the manus, and the digit tips were rounded (Figure 3I; compare this to Figure 3H).

### Claw development in *T. annularis*

**Figure 4:**
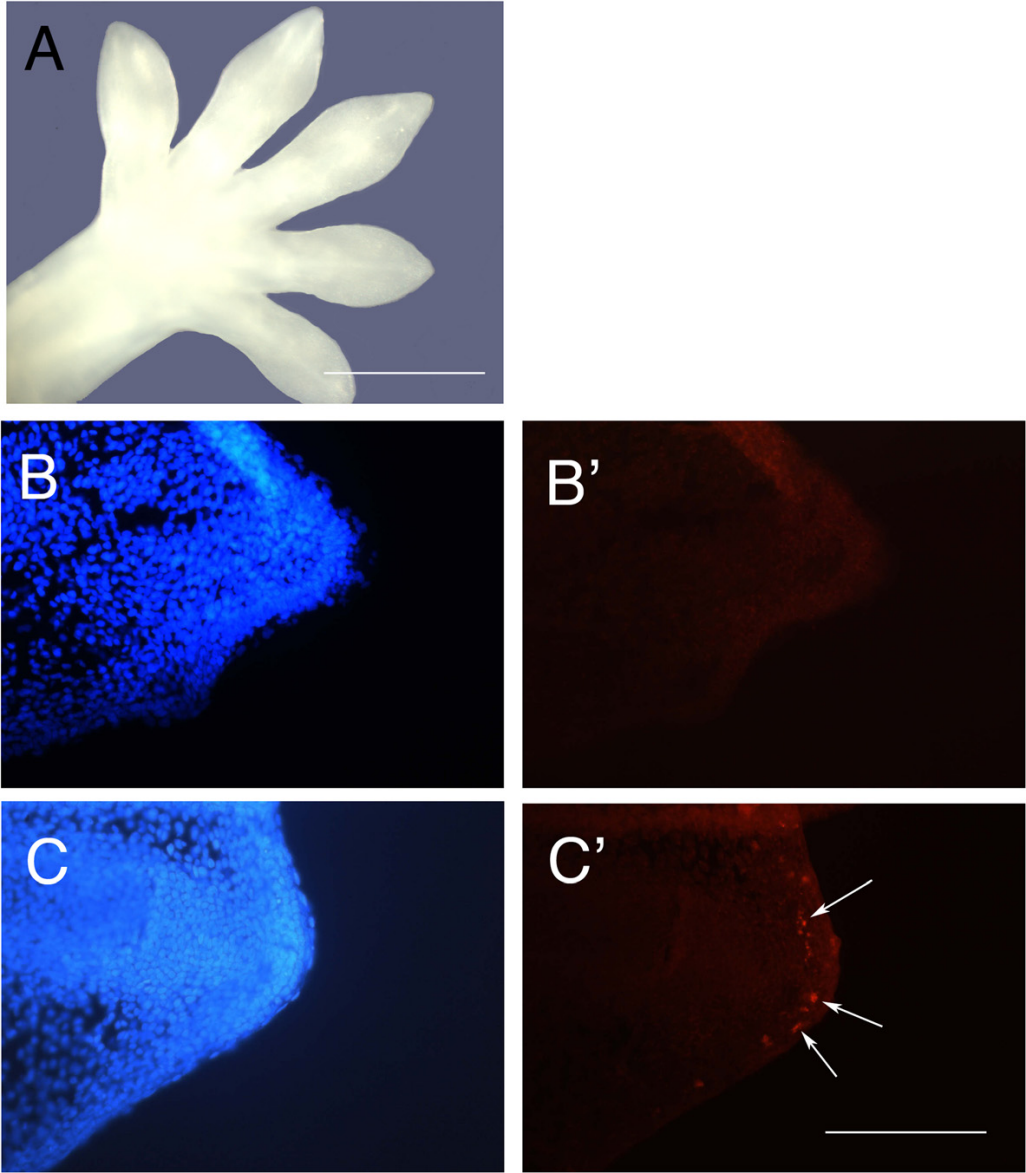
**Evidence of programmed cell death during the early stages of the development of unclawed manual digits of** ***Tarentola annularis*** **. (A)** Dorsal view of the entire left manus in the early part of stage 35 of development, a time at which all digits appear identical. **(B,B’,C,C’)** Details of the tip of manual digits II and III in the early part of stage 35 of development. DAPI staining **(B,C)** and activated caspase-3 staining (red spots) **(B’,C’)** show no evidence of apoptosis in the clawed digit **(B’)**, but concentration of apoptotic activity in the mesenchyme underlying the surface epithelium at the tip of the unclawed digit **(C’)** (arrows). Scale bar 1 mm for **(A)** and 100 μm for **(B,B’,C,C’)**.

To determine whether any differences were already present in the distal tips of the digits prior to any visible evidence of the anatomical differentiation of the claws, we examined early stage 35, prior to the initiation of claws (Figure 4A). At this stage, all digits terminate in a point at the distal end of the expanding adhesive pad, and the condensing mesenchyme of the future phalanx lies just under the tip epithelium (Figure 4B,C). At this stage, elevated levels of cell death were evidenced by activated caspase-3 present in the mesenchyme underlying the epithelium of the unclawed and reduced-clawed digits (Figure 4C’) relative to the clawed digits (Figure 4B’). Cell death was not observed in the developing cartilage, indicating that apoptosis may not explain the later size difference that is evident between the ungual phalanges of the clawed and unclawed/reduced-clawed digits (Figure 1L,M,N,O). Our results suggest that cell death does play a role in shaping the tip of unclawed/reduced-clawed digits, although other developmental processes are also likely to be involved.

**Figure 5:**
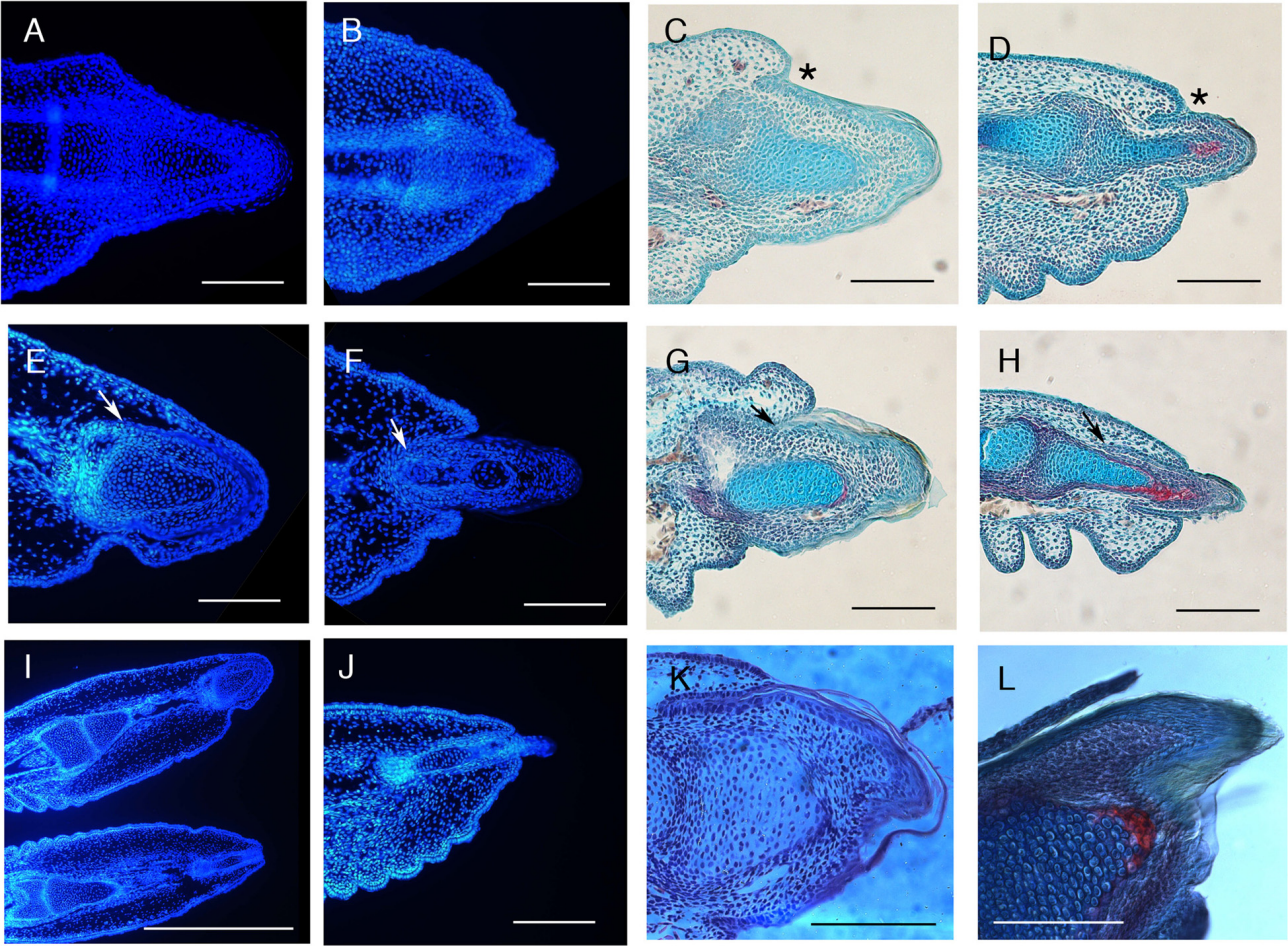
**Histology of claw development in** ***Tarentola annularis*** **.** Panels to the left **(A,B,E,F,I,J)** depict DAPI staining for nuclei in the developing claws in frontal section. Panels to the right **(C,D,G,H,K,L)** depict trichrome staining of developing claws in longitudinal section. Panels **(A,C,E,G,K)** and **(L)** are sections of clawed digits; panels **(B,D,F,H)** and **(J)** are sections of unclawed/reduced-clawed digits; panel **(I)** shows adjacent clawed (digit III, above) and unclawed (digit II, below) digits. **(A,B)** Early stage 35 (manual digits). **(C,D)** Late stage 35 (manual digits). Asterisk indicates the location of the fold in the epidermis dorsal to the claw. **(E-J)** Late stage 36 (pedal digits). In **(E)** and **(H)**, the arrow indicates the location of the apex of the hinge between the developing claw and the overlying dorsal scale. **(K,L)** Keratinized claw (manual digit III) at early and late stage 37, respectively. **(C,D)** Asterisk indicates position of claw fold. **(E-H)** Arrow indicates base of hinge between the claw and the dorsal scale. Scale bar 100 μm for **(A-H,J)**, 500 μm for **(I)** and 100 μm for **(K,L)**.

By late stage 35, a thickening of the epithelium was evident covering the tips of both the clawed (Figure 5A) and unclawed/reduced-clawed (Figure 5B) digits, with the distalmost phalanx lying along the midline axis of the distal digital extension. At this stage of development, the clawed digits already displayed a larger tip (Figure 5A,B), agreeing with the earlier observation that differences between the clawed and unclawed/reduced-clawed digits are present before the anatomical initiation of claw development (Figure 4B,B’,C,C’). By stage 36, folds representing the early stages of scale differentiation were evident, and an epithelial fold was present at the proximal end of the terminal epithelial thickening (Figure 5C,D). At this stage, the distalmost phalanx of the clawed digits (Figure 5C) is much taller and more robust than that of the unclawed/reduced-clawed digits (Figure 5D), and the shape of the forming claw sheath reflected this difference. Thus although claws do form on all digits, they are not equivalent.

By the end of stage 36, the digital scales were more pronounced, and the distalmost mid-sagittal dorsal scale had grown over the base of the claw epithelium, creating a hinge (Figure 5G,H). The difference between the distalmost phalanx of the clawed and unclawed/reduced-clawed digits was even more pronounced at this stage (Figure 5E-J). The nail-like scale that overlies the unclawed/reduced-clawed digit tips (Figure 1B,C,F) was evident as a large, distallyextensive developing scale at stage 36 of development (Figure 5H). Interestingly, the distalmost region of the ungual phalanx of the unclawed/reduced-clawed digits (Figure 5H) initiated endochondral ossification earlier than that of the clawed digits (Figure 5G,K). Ossification of the tip of the distalmost region of the ungual phalanx of the clawed digits was not observed until the latter part of stage 37 (Figure 5L).

### Reduction and loss of claws through apoptosis and proliferative changes

**Figure 6:**
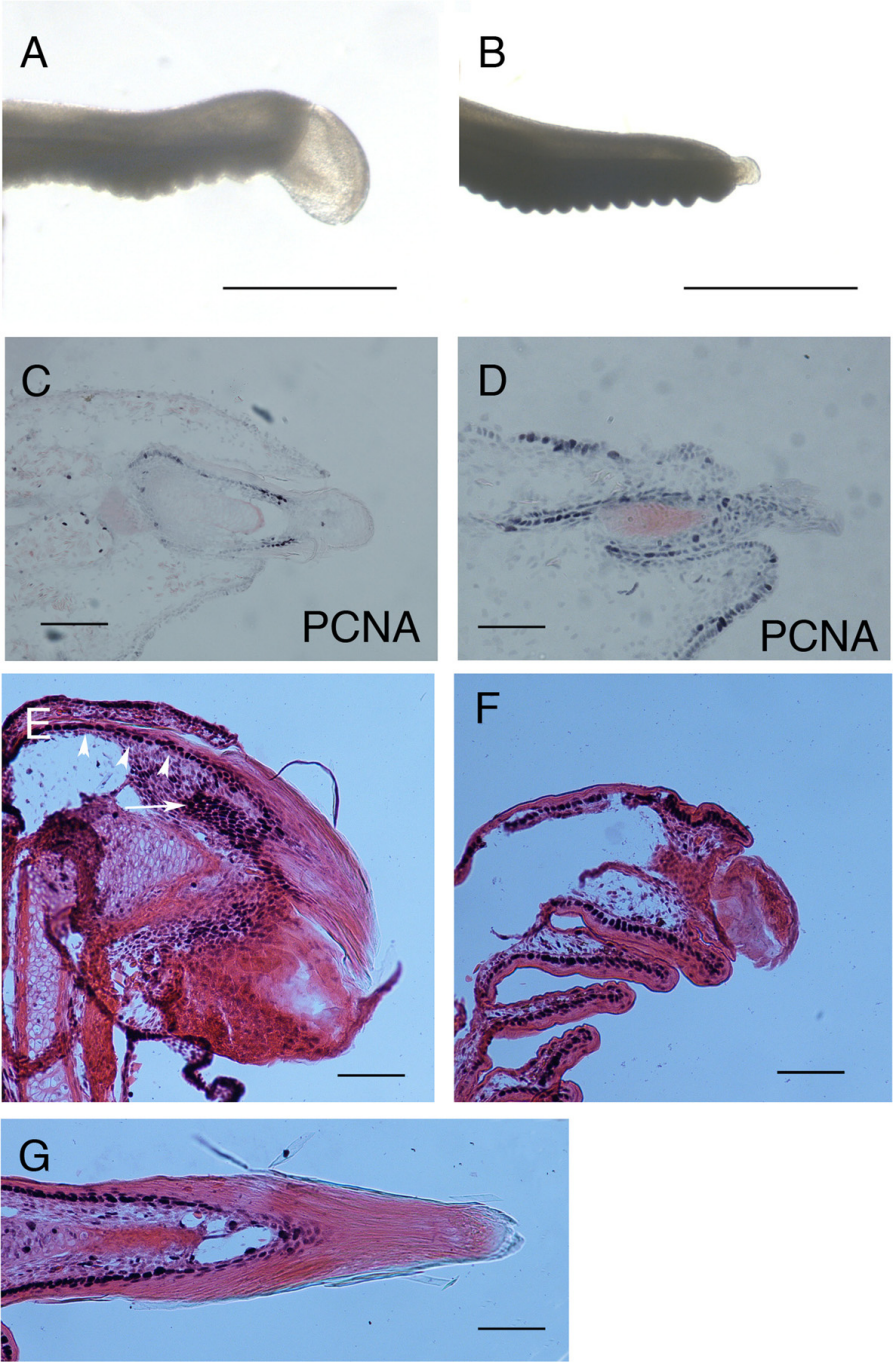
**Cellular proliferation in the developing claws of** ***Tarentola annularis*** **. (A-D)** Pedal claws at late stage 36 of development. **(A)** digit III, **(B)** digit II. **(C-G)** PCNA staining. (**C**,**D** - longitudinal sections) Weak eosin counterstaining in late stage 36. **(E-G)** Strong eosin counterstaining in stage 38. Cellular proliferation (**C**,**D**, black dots) is evident along the claw epithelium in both clawed **(C)** and unclawed **(D)** digits. Proliferation is evident at the base of claw **(E)** in the clawed digit (arrow) and throughout the dorsal claw epithelium (arrowheads). Cellular proliferation in the unclawed digit (**F** - longitudinal section) is confined to the cells underlying the surface epithelium, although it is abundantly expressed in the epithelium of the developing adhesive plates (arrows). **(G)** Frontal section through the claw of a pedal clawed digit (III) showing the proliferating epithelium lining the inner surface of the claw sheath, adjacent to the underlying ungual phalanx. Scale bar 500 μm for **(A,B)** and 100 μm for **(C-G)**.

As we identified that claws initiated on all (adult clawed, reduced-clawed, and clawless) digits (Figure 5), the question arose as to whether the later reduction or loss of the claws on digits I, II, and V resulted from changes in rates of cellular proliferation and/or increases in programmed cell death. We therefore investigated the regression of the claws from late stage 36 onwards (Figure 6A,B). Consistent with previous studies of cellular proliferation in lizards, intense rates of proliferation were characteristic of the developing claw sheath epithelium lying adjacent to the ungual phalanx (Figure 6C). Interestingly, high levels of proliferation were associated with the claw epithelium of both clawed (Figure 6C) and clawless/reduced-clawed (Figure 6D) digits, even though the claw sheath on the clawless/reduced-clawed digits had begun to disappear by this stage (compare Figure 6A,B). By late stage 37, however, a clear difference was evident between clawed and clawless/reduced-clawed digits. High levels of proliferation were evident at the base of the claw and along the epithelium on the deep aspect of the unguis adjacent to the keratinous claw in the clawed digits (Figure 6E,G). In contrast, no proliferation was observed in these locations in the clawless/reduced-clawed digits at this developmental stage (Figure 6F). High levels of proliferation, however, were evident in the basal cells of the epithelium of the subdigital lamellae (Figure 6F), confirming the ability to detect proliferative activity in these samples. It appears, therefore, that although changes in proliferation are not responsible for the initial reduction of the claw on unclawed/reduced-clawed digits, they may play a role in the subsequent failure of further growth.

**Figure 7:**
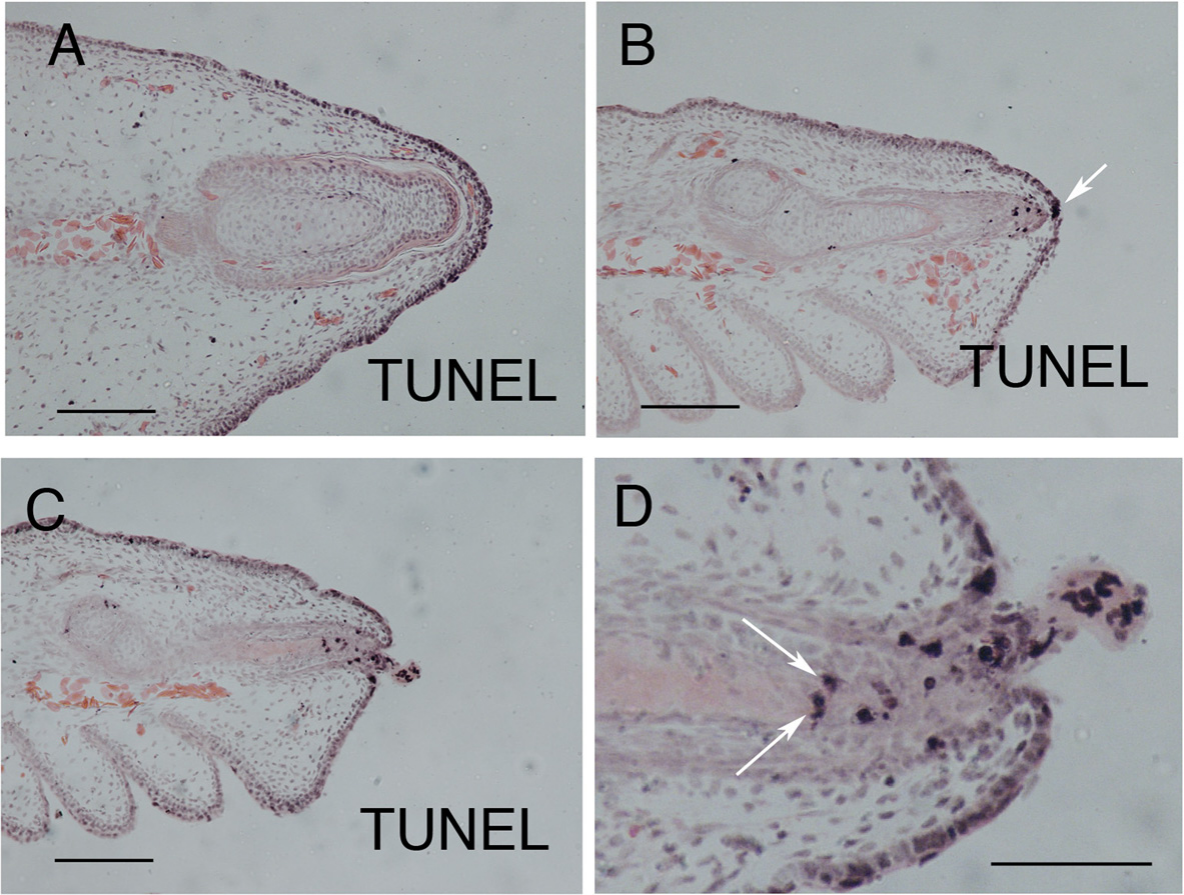
**Cell death in the regressing claws of** ***Tarentola annularis*** **.** TUNEL stainining for apoptotic cells, which appear black, at stage 36 of development. **(A)** In the clawed pedal digit III apoptotic cells are associated only with the overlying surface epithelium. In the unclawed pedal digit II, **(B,** **C)** positively-reacting cells are located in the developing claw and in the overlying epithelium (arrow in **B**). **(D)** A higher power view of the region depicted in panel **(C)** shows positively reacting cells in both the claw sheath and the underlying ungual phalanx (arrows). Scale bars, 100 μm.

Using TUNEL, we also investigated apoptosis at stage 36. Large numbers of TUNEL positive cells were associated with the developing claws on the unclawed/reduced-clawed digits, whereas no positive cells were evident in the forming claw on the clawed digits (Figure 7). In unclawed/reduced-clawed digits, positive cells were associated with the cells of the forming claw epithelium and mesenchyme, indicating a central role for apoptosis in removal and reduction of the claw itself (Figure 7B,C). Cell death was also observed at the distal tip of the ungual phalanx (Figure 7D). Cell death in this phalanx may explain its reduced size and altered form on the unclawed/reduced-clawed digits. It may, however, also be associated with the early onset of endochondral ossification that occurs in this region in these digits as chondrocyte cell death plays an active part in this process [27].

## Discussion

Our investigations reveal that in *Tarentola annularis*, claws initially develop on all five digits of the manus and pes, that even at their earliest stages of detection, there are differences in cellular activity in the distal tips of the digits, and that the claws on digits I, II, and V regress during the later stages of development. Reduction and loss of these claws appears to result from a combination of several processes. Even before the claw sheaths begin to differentiate from the surface epithelium of the digit tips, differences were evident in the shape of the distalmost phalanges, associated with elevated levels of cell death in the mesenchyme of those digits in which the claw will be reduced or lost. This greater intensity of cell death may be responsible for the reduced size and altered form of the terminal phalanx of these digits as development progresses. Once the claw sheath had initiated, the position of the epithelial fold differed between the clawed and clawless/reduced-clawed digits. The groove of the fold was located more proximally in the clawed digits, and the enlarged claw extended distally from this. In the clawless/reduced-clawed digits, the fold gives rise to a nail-like scale in the dorsal midline (Figure 1B,C,F) that roofs and internalizes the majority of the elongated ungual phalanx and the majority of the reduced claw sheath where this persists.

High rates of proliferation were associated with the initial development of the epithelium of both the clawed and clawless/reduced-clawed digits, but proliferation was diminished in the clawless/reduced claw epithelium during later stages. High levels of apoptosis were observed in the unclawed/reduced-clawed digits, with cells being removed from within and around the forming claw sheath. Given the large number of dying cells observed, it is likely that apoptosis is the central mechanism leading to reduction and loss of the developing claws.

In the clawed digits, at the same stage (stage 36), very little endochondral ossification was evident in the ungual phalanx, which later developed into a large distal element supporting the fully developed claw sheath on digits III and IV. In contrast, the terminal phalanx of the clawless/reduced-clawed digits was much smaller and the cartilage had been replaced by bone at its distal tip. Such early onset of endochondral ossification may be associated with the relatively reduced size of this skeletal element on the unclawed/reduced-clawed digits. Apoptosis was also observed in the terminal phalanx associated with the disappearing claw. Cell death in this region could be responsible for further thinning and attenuation of the ungual phalanx, or it could be a consequence of the early endochondral ossification [27]. A few apoptotic cells were associated with the large terminal phalanx of the clawed digits at later stages as these started to undergo ossification, but these were mainly associated with the perichondrium. It is therefore unclear whether apoptosis or early endochondral ossification is the major driver of the reduction of this phalanx.

Apoptosis is a well-known mechanism for bringing about the elimination or reduction of structures during development. For example, in tooth development, apoptosis is associated with the removal of vistigial tooth germs, enamel knot signaling centers, and loss of ameloblasts [19,28]. During digit development, the interdigital webbing is removed as a result of apoptosis, allowing the digital rays to be freed [2]. If this process fails or is suppressed, syndactyly occurs [29,30], which is a natural occurrence in taxa that exhibit webbed digits [31]. Recently, it has been shown that apoptosis is responsible for the loss of whole digits in the horse, camel, and three-toed jerboa, although not for digit reduction in the pig [20]. In the case of the jerboa, the loss of digits appears to have co-opted the pathways normally used to remove interdigital tissue in the limb, as the transcription factor *Msx2* overlaps with cell death in both cases, and *Bmp4* is upregulated [20]. Whether a similar correlation of *Bmp4, Msx2*, and cell death occurs in the regressing gecko claw digits provides an interesting avenue for future exploration, as *Msx2* has been shown to affect the length of murine nails and claws by suppressing proliferation of germinal epithelium [32]. In skinks, changes in the timing of *Shh* expression have been highlighted as leading to digit loss, with reduced proliferation of digit mesenchyme resulting in digit regression [23]. Study of the molecular signals involved in gecko claw development and regression are therefore necessary for identifying whether the same conserved pathways (*Bmps*, *Shh*, *Fgfs*) are utilized to sculpt different aspects of the limb at different stages of development.

The relationship between the phalanx and the overlying claw sheath is unclear. In the process of claw reduction and loss during development, both structures appear to be involved, but whether one triggers changes in the other is unknown. It seems likely that the initially smaller protrusion at the tip of the unclawed/reduced-clawed digits constrains the region that will develop into the claw, and the thinner, elongate form of the terminal phalanx leads to the formation of a narrower claw sheath that caps this skeletal element. The resulting smaller claw sheath either persists in an abbreviated form in the pes or is lost entirely in the manus through further apoptosis, and these events are accompanied by an arrest of cell proliferation.

The digit field is formed by a small apical dermal-epidermal region at the tip of the limb bud [33], with an active dermis in contact with a thickened epidermis. After the ungual phalanx begins development, the terminal epidermis commences to thicken, yielding the first sign of the claw sheath. A possible influence on the process of claw sheath morphogenesis by the perichondral cells of the ungual phalanx was advanced by Alibardi [9]. He proposed that close contact between the epithelium and the ungual phalangeal chondrocytes might trigger the earlier production of keratins (tough β-keratins on the dorsal side of the claw and pliable α-keratins on its ventral side) in the forming claw sheaths than in the other scales of the digits. The keratinocytes of the developing epidermis of the claw sheath lie in direct contact with the mesenchymal cells of the ungual phalanx [9].

Like those of reptiles, mammalian claws derive from a thickening of the embryonic epidermis of the dorsal tips of digits. However, in mammals, the growth of claws and nails occurs by the continuous production of new keratinocytes from a proximal germinal matrix [7,10,34-39]. This has been confirmed by following digit regeneration in the mouse [40]. Proliferation in mammals is confined to the base of the developing nail/claw. Our results accord with the findings of Alibardi [8] that proliferation in geckos occurs throughout the claw epithelium. Significant differences therefore exist between claw development in reptiles and mammals.

Our prediction that all digits would exhibit identical early stages of claw formation and that there would be regression of claw sheath expression in those digits that ultimately exhibit only minute claws or their complete absence is generally supported. However, it is evident that subtle differences between digits that will ultimately bear fullydeveloped claws and those on which claws will be reduced or absent are evident from the earliest stages of detection. Also supported is our suggestion that apoptosis plays an important role in the reduction and elimination of the claw sheath rudiments. Our prediction that apoptosis is accompanied by a reduction in proliferation of generative cells is also confirmed. Differential developmental timing of a common set of processes leads to a variety of outcomes and also results in the reshaping of the ungual phalanx and its associated sheath in those digits displaying claw reduction or loss.

Robust, curved claws that cap the ends of the digits of the manus and pes constitute the primitive condition for lizards. Among lizards exhibiting fully developed (non-reduced) limbs, only in the Gekkota is the reduction and absence of claws prevalent [11]. This reduction and loss is always associated with taxa that possess a subdigital adhesive system (including lineages within these that have secondarily lost this system [41]). Claw reduction and loss has occurred independently at least ten times within the Gekkota (see Introduction). Furthermore, reduction and loss of claws is generally associated with elongation of the ungual phalanx. In the *Gekko*, *Homopholis*, *Hemidactylus* (Gekkonidae), and *Pseudothecadactylus* (Diplodactylidae) clades, this is restricted to digit I of the manus and pes. It has been argued that this is associated with a trade-off of functional capabilities of digit I [17], and that claw reduction/loss and the reconfiguration of the ungual phalanx is implicated in increasing the available subdigital adhesive pad area.

The function of the needle-like vestigial claws (especially on the pedal digits) that are retained in *Tarentola* and other taxa remains unknown, but their sexually dimorphic form (being more robust in females) suggests that they may be involved in the manipulation and placement of eggs at the time of laying (see above; [14,15]).

## Conclusions

The mechanisms underlying claw reduction and loss in *Tarentola annularis*, as presented in this contribution, may underlie claw reduction in other lineages. This must remain tentative, however, because for mammals, it has been shown that different mechanisms yield ostensibly the same general pattern of digit loss [20]. Differential claw and terminal phalanx expression among digits is seemingly related to the surface area of the subdigital adhesive pads that can be carried by particular digits and thus can be regarded as an evolutionary trade-off between functional demands. On certain digits, reduced claws may retain some but not all of their original functional roles; and in some taxa, this differentiation of roles is manifested in sexual dimorphism. Since *Tarentola* exhibits full expression, reduction, and complete loss of claws in the same individual, it constitutes an excellent vehicle for investigating differential developmental mechanisms that lead to evolutionary reduction and loss of structures.

*Tarentola* is unusual in that it retains fully developed claws only on digits III and IV of the manus and pes. Thus, digits I, II, and V likely benefit from enhanced adhesive pad area in association with the reduction or loss of the claws and the elongation of the ungual phalanges. The retention of fully developed claws on digits III and IV likely assists in attachment to the rocky surfaces typically occupied by members of this genus [42]. More needs to be known about the functional role of the individual digits in various activities, including locomotion on various surfaces, and during egg deposition.

## Additional file

Additional file 1: Table S1. Embryonic stages and identity and number of digits of *Tarentola annularis* (Khannoon, unpublished) used in the various procedures (PCNA, DAPI, caspase-3, TUNEL and SEM) in this study. Morphological staging follows the criteria set out by Hamburger and Hamilton [24]. Stage duration as day post-oviposition (dpo) is given.

## Abbreviations

BAMBI: bone morphogenetic protein and activin membrane-bound inhibitor
Bmp: bone morphogenetic protein
BSA: bovine serum albumin
DAB: 3,3′-diaminobenzidine
DAPI: 4′,6-diamidino-2-phenylindole
dpo: day post-oviposition
DPX: di-n-butyl phthalate in xylene
EDTA: ethylenediaminetetraacetic acid
Fgf: fibroblast growth factor
Msx2: homeobox transcription *factor* muscle segment homeobox 2
PBS: phosphate-buffered saline
PCNA: proliferating cell nuclear antigen
RT: room temperature
Shh: sonic hedgehog
TDT: terminal deoxynucleotidyl transferase
TUNEL: terminal deoxynucleotidyl transferase dUTP nick end labeling
