# Supplementary material for: Developmental mechanisms underlying differential claw expression in the autopodia of geckos
Source: EvoDevo. 2015 Apr 10;6:8. doi: 10.1186/s13227-015-0003-9 (PMC4397723; doi:10.1186/s13227-015-0003-9)
Supplement: Additional file 1: Table S1. — Embryonic stages and identity and number of digits of Tarentola annularis (Khannoon, unpublished) used in the various procedures (PCNA, DAPI, caspase-3, TUNEL and SEM) in this study. Morphological staging follows the criteria set out by Hamburger and Hamilton [24]. Stage duration as day post-oviposition (dpo) is given. [file 13227_2015_3_MOESM1_ESM.pdf]

Additional Table 1. Embryonic stages and identity and number of digits of *Tarentola annularis* (Khannoon, unpublished) used in the various procedures (PCNA, DAPI, Caspase-3, TUNEL and SEM) in this study. Morphological staging follows the criteria set out by Hamburger and Hamilton [24]. Stage duration as day post-oviposition (dpo) is given.

**Stage**	**Number of animals used**	**Days *in ovo*** (dpo)	**Autopodium used**	**Digits used**	**Technique** (for each digit, minimum of 5 samples were subjected to each treatment, except for SEM)
35	13	24-30	left manus and pes	all digits	DAPI&Caspase-3 (4x digit II, 6x digit III). Trichrome (5x digit II, 7x digit III). SEM (2x digit I, 2x digit IV, 2x digit V). DAPI (5x digit II, 4x digit III)
36	9	31-34	left manus and pes	I, II, III&V	SEM (2x digit I, 2x digit III, 2x digit V). Trichrome (5x digit II, 7x digit III). DAPI (4x digit II, 4x digit III). PCNA (7x digit II, 7x digit III). TUNEL (7x digit II, 7xdigit III)
37	7	35-40	left manus and pes	III	Trichrome (5x digit III), SEM (2x digit III),
38	4	41-51	left manus and pes	II, III, IV &V	PCNA (2x digit II, 2x digit III). SEM (2x digit IV, 2x digit V)
